# Relocation, Health, and Physical Activity Engagement in Senior Living Community Residents

**DOI:** 10.1093/geroni/igaf122.2749

**Published:** 2025-12-31

**Authors:** Chiung-ju Liu, Gabriella Ulloa, Kelly Leal

**Affiliations:** University of Florida College of Public Health and Health Professions, Gainesville, Florida, United States; University of Florida College of Public Health and Health Professions, Gainesville, Florida, United States; University of Florida College of Public Health and Health Professions, Gainesville, Florida, United States

## Abstract

Senior living communities offer supportive environments to promote healthy aging. However, little is known about the health status and the degree of physical activity engagement to support health in the community residents. The purpose of this study was to describe the health status and how the health status related to reasons for relocation and the use of fitness amenities. Residents who had lived in a senior living community for over 3 months in two cities of Florida were invited to complete a survey between December 2023 and November 2024. Participants answered questions about relocation reasons, fitness amenity utilization, frailty (Tilburg Frailty Indicator), sarcopenia (Strength, Assistance with walking, Rise from a chair, Climb stairs, and Falls), and cognitive changes (Ascertain Dementia 8-item Informant Questionnaire). A total of 145 residents responded to the survey (Mean age = 80 years; Female n = 104). Results indicate that 28% of respondents have frailty, 20% have sarcopenia, and 22% are at risk of cognitive impairment. Residents who relocated due to declining health (n = 25) reported greater degrees of frailty, sarcopenia, and cognitive changes relative to those due to non-health reasons (Mann-Whitney U Test, all ps < .05). Additionally, residents who used the fitness amenities more often is associated with less frailty and sarcopenia (Spearman’s Rho Test, both ps < .01). Study findings suggested that residents with a relocation reason related to declining health may be at risk of poor health. Frequent use of community fitness amenities correlates with better physical health.

